# Combined Training Intervention Targeting Medical and Nursing Staff Reduces Ciprofloxacin Use and Events of Urinary Tract Infection

**DOI:** 10.1155/2022/2474242

**Published:** 2022-04-11

**Authors:** Johannes Forster, Petra Schulze, Claudia Burger, Manuel Krone, Ulrich Vogel, Güzin Surat

**Affiliations:** ^1^Institute for Hygiene und Microbiology, University of Würzburg, Würzburg, Germany; ^2^Division of Infectious Diseases, Department of Internal Medicine II, University Hospital of Wuerzburg, Würzburg, Germany; ^3^Pharmacy, University Hospital of Würzburg, Würzburg, Germany; ^4^Department of Infection Control and Antimicrobial Stewardship, University Hospital of Würzburg, Würzburg, Germany

## Abstract

Inappropriate diagnosis of urinary tract infections (UTI) contributes to antimicrobial overuse. A combined training intervention for medical and nursing staff mainly addressing the analytic process reduced UTI events (9.20 vs. 7.36 per 1000 PD, −20.0%, *p* = 0.003) and the utilization rate of ciprofloxacin (11.6 vs. 3.5, −69.6 *p* = 0.001) in a Bavarian University Hospital. Combined training intervention—as part of an antibiotic stewardship program—can be effective in avoiding unnecessary urinalysis and reducing antibiotic consumption.

## 1. Introduction

Urinary tract infections (UTIs) are the third most common type of healthcare-associated infections in Germany and the fifth most common in the United states accounting for a considerable amount of in-hospital antibiotic consumption [1, 2]. UTIs have been successfully addressed by antibiotic stewardship programs (ASP) [3], and the current IDSA guideline on antimicrobial stewardship strategies highlights the challenge of preventing antibiotic treatment for asymptomatic bacteriuria (ASB). Fluoroquinolones were commonly used to treat UTI in the outpatient setting, but due to severe adverse events and high resistance rates among *E. coli*, they are a major target of ASPs [4]. Especially, the adverse events prompted to national and international safety warnings discouraging the empiric treatment of mild/moderate infections with fluoroquinolones including uncomplicated urinary tract infections [5, 6]. ASPs put emphasis on improving the quality of antibiotic prescribing; interventions targeting the analytic process are particularly suitable to avoid analyzing and reporting and hence triggering unnecessary treatment of ASB.

The analytic process consists of preanalytics (indication, sampling, and transport), analytics (species identification and antibiotic susceptibility testing), and postanalytics (interpretation of findings in the clinical context). Analytics and early stages of postanalytics (i.e., selective reporting) are subjects to the laboratory process and may therefore be controlled straightforwardly [7]. However, it takes more effort to modify pre and postanalytical procedures, as they are executed outside the laboratories by healthcare staff.

Education can change behavior of medical personnel. It is a core element of ASPs and has the power to influence all persons responsible in the pre and postanalytical processes [8]. Nursing staff was recognized as a potential player in the implementation of ASP, and nurse-driven antibiotic stewardship practices (need for urine cultures and ensuring the proper culturing technique) were identified [9]. Potential effective measures of ASPs to partner with nursing staff need to be investigated. This study evaluates the effect of a combined training intervention for medical and nursing staff on diagnosis and treatment of urinary tract infections.

## 2. Intervention/Methods

This educational intervention was carried out between January and June 2019 at Wuerzburg University Hospital (UKW), a 1430 beds tertiary care hospital in Bavaria, Germany. We identified both medical and nursing staff as target groups and conducted training on pre and postanalytics of urinary tract infections.

For physicians, particular emphasis was placed on indication for UTI diagnostics, diagnostic criteria of UTI and management of ASB, and antibiotic therapy of UTI. Training was held in 14 departments (cardiothoracic surgery, dermatology, emergency, gynecology, internal medicine, intensive care, neurology, neurosurgery, otolaryngology, ophthalmology, orthopedics, oral and maxillofacial surgery, radiology/radiation therapy, and general/vascular surgery). Nursing staff was addressed in monthly held educational sessions. Nursing staff at UKW is advised to participate in these sessions once per year. Attendance of training was documented for 48% of nurses in 2019. Focus was set on indications for UTI diagnostics (avoid urinalysis in asymptomatic individuals and avoid urine dipstick from indwelling catheters), sampling (hierarchy of sampling methods, hygienic sampling procedure, and avoid urine dip slide for culture), and transport (immediate transfer to the laboratory or storing at 4° overnight, information on logistics and laboratory information system).

To quantify the impact of the training intervention, we assessed the following indicators before (July–October 2018) and after intervention (July–October 2019): access frequency to the local guideline for UTI, number of urinalysis, number of urine culture, UTI events, and antibiotic use rate (AUR) of ciprofloxacin, fosfomycin and nitrofurantoin, which were appraised on a monthly basis.

In Germany, hospital revenues are standardized by diagnosis related groups (DRG). UTI events were requested from the hospitals controlling based on ICD-10 diagnosis N39.0 (urinary tract infection).

The recommendation of the local guidelines is based on local antibiotic resistance. Results of susceptibility testing of urinary samples at UKW in 2019 are provided in Supplementary Table 1.

IBM SPSS® Statistics version 24 (Armonk, NY, USA) and Microsoft® Excel® 2016 (Redmond, WA, USA) were used for statistical analysis. No variable showed a significant aberrance from normal distribution using the Shapiro–Wilk test. Welch's *t*-test was used on UTI diagnosis (significantly different variances in Levene's test) and Student's *t*-test on all other indicators (no significantly different variances). Two-tailed significance level *α* was set to 0.05.

## 3. Results

Access frequency to the hospital guideline for UTI almost doubled after intervention. While from July to October 2018, it was accessed 44 times, between July and October 2019, and staff consulted the document 86 times (+96.0%, *p* = 0.09, Table 1). Access raised to a peak one month after intervention was completed and remained steady.

The number of urinalysis decreased slightly after intervention, but did not reach statistical significance (108.5 vs. 103.2 per 1000 PD, −4.9%, *p* = 0.307). The number urine cultures from indwelling catheters increased from 4.29 to 5.11 per 1000 PD (+19.0% *p* = 0.038). This increase in appropriate UTI diagnostic leads to a significant decrease in diagnosed UTIs, classified on the basis of ICD-10, after intervention (9.20 vs. 7.36 per 1000 PD, −20.0%, *p* = 0.003).

**Figure 1:**
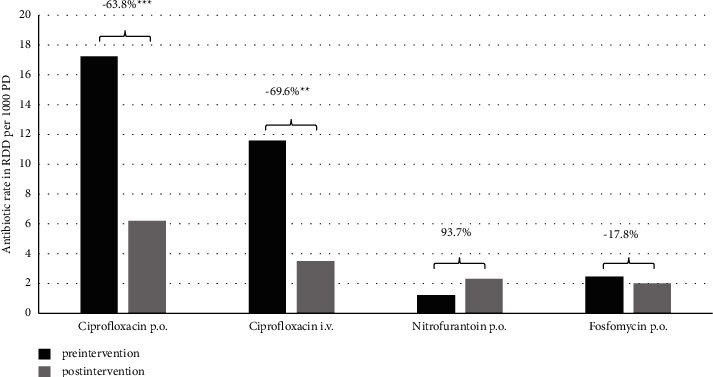
Antibiotic consumption of ciprofloxacin, fosfomycin, and nitrofurantoin. RDD, recommended daily doses; PD, patient days. ^∗^Statistical significance, ^*∗∗∗*^*p* < 0.001, ^*∗∗*^*p* < 0.01.

Regarding antibiotic choice, the main objective of the training intervention—reduction of the AUR of ciprofloxacin—was achieved as its use declined significantly. Oral ciprofloxacin was reduced from 17.2 to 6.2 RDD per 1000 PD (−63.8%, *p* < 0.001) and use of parenteral ciprofloxacin declined from 11.6 to 3.5 RDD per 1000 PD (-69.6, *p* < 0.001) after intervention. Total ciprofloxacin AUR was reduced from 11.6 to 3.5 RDD per 1000 PD (−69.6 *p* = 0.001, Figure 1).

In parallel with the reduction of ciprofloxacin, the AUR of fosfomycin decreased slightly (2.4 vs. 2.0 per 1000 PD, −17.8%, *p* = 0.44). At the same time, AUR of nitrofurantoin doubled, but as a result of the low initial level, this did not reach statistical significance (1.2 vs. 2.3 per 1000 PD, 93.7%, *p* = 0.13).

## 4. Discussion

The targets of the training intervention presented herein were the frequency of the local guideline, the number of urinalysis and of diagnosed UTIs, and the AUR of ciprofloxacin.

In this study, the access to the local guideline increases significantly after intervention. This is important as facility-specific treatment guidelines for antibiotic prescribing have been positively evaluated in inpatient and outpatient settings and are among the priority intervention ASPs [10, 11]. However, impact on clinical management is unclear and it is therefore considered indirect and hence the soft indicator.

The overall number of urinalysis did not decrease after intervention. A reason might be that apart from diagnosis of UTI, other parameters of this integrated test (albumin, glucose, and bilirubin) are in routine use. In a study by Lim et al. on nurses` knowledge, perception, and practice around urinalysis, performing urinalysis on all new admissions was identified as routine practice by 89% and 35% of patients with indwelling catheters [12]. However, due to predictable colonization, the inappropriateness of urinalysis from indwelling catheters, especially in the absence of clinical symptoms of UTI, is key aspect of ASP and a focus of our training presentations [13, 14]. In the last three years, the request for urine cultures from indwelling catheters rose heavily at UKW. Although our intervention did not reverse this trend, the increase was mitigated. It has to be stressed that urinalysis is a useful tool to rule out UTI if an accurate interpretation of the results is provided.

According to national and international guidelines, the triad of clinical symptoms, pyuria, and cultural pathogen detection has to be fulfilled to diagnose UTI [15, 16]. Complying with these diagnostic criteria prevents from overtreatment. Therefore, in the training of the medical staff, we focused on postanalytics, specifically the prevention of treatment solely based on bacterial growth or nitrite in absence of clinical signs suggestive for UTI. After intervention, the number of microbiological urinalysis increased, while the number of diagnosed UTI declined. This suggests gain of competence in the interpretation of results (postanalytics) of the medical staff by the training provided. Our results are consistent with findings by Lee et al. who performed a three-month training intervention in a Canadian long-term care facility. In their study, the primary outcome—the number of residents who received inappropriate antibiotic treatment for asymptomatic bacteriuria—was significantly reduced from 90.0% to 62.9% [17].

Regarding antibiotic choice of treatment of UTI, the key message of our training intervention was the recommendation against the use of fluoroquinolones. AUR rates of intravenous and oral ciprofloxacin dropped significantly (−69.6% and −63.8%). The choice of agent was addressed in the training events, and alternatives to fluoroquinolones were advocated for complicated and uncomplicated UTI. For uncomplicated UTI, clinicians preferably chose nitrofurantoin (+93.7%) over fosfomycin (−17.8%). One reason for the preference of nitrofurantoin over fosfomycin might be that the latter is considered as a last-resort antibiotic even when it comes to uncomplicated urinary tract infections. The advantage of nitrofurantoin in the treatment of uncomplicated UTI has recently been demonstrated, although the known adverse effects, e.g., liver injuries or interstitial pneumonitis, should be not lost out of focus [18].

Our study has important limitations. At UKW, electronic patient record is not available for all wards. Therefore, diagnosis-related assessment of AUR was not possible, and AUR of ciprofloxacin includes prescription for other indications than UTI. The intensified warning of the German Federal Institute of Drugs and Medical Devices (Bundesinstitut für Arzneimittel und Medizinprodukte, BfArM) in April 2019 on risks of adverse events caused by fluoroquinolones may have influenced physicians in their prescribing behavior. Furthermore, we did not evaluate the knowledge pre and postintervention, neither did we measure the satisfaction scale of the target group. Reduction of AUR of fluoroquinolones is a main target of our ASP and rates dropped from 10.5% to 40.7% per year since 2016 (data not shown). Our intervention reinforced the efforts of reducing AUR of ciprofloxacin and so did other ongoing measures as ward rounds, local guidelines, and selective antibiotic reporting. Last, owing to its retrospective design, the study was not controlled for other factors that might have influenced the outcome.

## 5. Conclusion

The implementation of a combined training intervention involving medical and nursing staff can be effective in curtailing unnecessary urinalysis, subsequent UTI events, and antibiotic consumption.

## Figures and Tables

**Table 1:** Differences in evaluated indicators pre and postinterventions.

	July–October 2018 (preintervention)	July–October 2019 (postintervention)	Difference (%)	*P*
Access to SOP (*n*)	44	86	+96.0	0.009
Urine dipstick tests (per 1000 PD)	108.5	103.2	−4.9	0.307
Urine cultures from indwelling catheter (per 1000 PD)	4.29	5.11	+19.0	0.038
Diagnosis UTI (per 1000 PD)	9.20	7.36	−20.0	0.003
Antibiotic consumption (RDD per 1000 PD)				
Ciprofloxacin p.o.	17.2	6.2	−63.8	<0.001
Ciprofloxacin i.v.	11.6	3.5	−69.6	0.001
Nitrofurantoin p.o.	1.2	2.3	+93.7	0.13
Fosfomycin p.o.	2.4	2.0	−17.8	0.44

SOP, standard operating procedure; PD, patient days; UTI, urinary tract infection.

## Data Availability

The data used to support the findings of this study are available from the corresponding author upon request.

## Supplementary Materials

Supplementary Materials Supplementary Table 1. Susceptibility testing of the five most prevalent pathogens of urinary tract infections at UKW (2019).
