# Supplementary material for: Combined Training Intervention Targeting Medical and Nursing Staff Reduces Ciprofloxacin Use and Events of Urinary Tract Infection
Source: Adv Urol. 2022 Apr 11;2022:2474242. doi: 10.1155/2022/2474242 (PMC9017541; doi:10.1155/2022/2474242)
Supplement: Supplementary Materials — Supplementary Table 1. Susceptibility testing of the five most prevalent pathogens of urinary tract infections at UKW (2019). [file 2474242.f1.docx]

Supplement 1: Susceptibility testing of the five most prevalent pathogens of urinary tract infections at UKW (2019).
